# A Study on the Efficacy of Drug-Mediated Moist Wound Healing Combined With Phototherapy in the Management of Facial Abrasion Scars

**DOI:** 10.7759/cureus.98427

**Published:** 2025-12-04

**Authors:** Hao Y Shan, Gang Y Jin, Gui H Wang, Yan Li

**Affiliations:** 1 Aesthetic Surgery, Huakang Dermatology Hospital, Fuyang, CHN; 2 Otorhinolaryngology, Xianghe County People's Hospital, Hebei Medical University, Langfang, CHN; 3 Plastic Surgery, Tongling Municipal Hospital, Anhui Medical University, Anhui, CHN; 4 Otorhinolaryngology, Shanghai Yida Hospital, Shanghai, CHN

**Keywords:** drug-moist, facial abrasion scars, likert scale, manchester scar scale, photoelectric therapy, scar appearance, wound healing

## Abstract

Objective

This study aimed to evaluate the efficacy of drug-mediated moist wound healing combined with phototherapy in the treatment of facial abrasion scars, with a focus on improving both clinical outcomes and patient satisfaction.

Methods

A total of 40 patients with facial abrasion scars were randomly assigned to two groups: the saline and iodophor solution group (control group) and the combined treatment group (receiving both drug-moist wound healing and photoelectric therapy). Wound healing time, scar appearance, and patient-reported outcomes were compared between the two groups.

Results

The results showed that the combined treatment group demonstrated significant advantages in promoting wound healing (t = 2.65; P < 0.05), improving scar appearance (t = 4.59, 7.48; P < 0.05), and enhancing patient satisfaction (t = 4.07, 6.87, 4.43; P < 0.05) compared to the control group.

Conclusion

These findings suggest that the combination of drug-moist wound healing and photoelectric therapy is a promising therapeutic approach for the management of facial abrasion scars. It can significantly shorten the wound healing time, improve scar appearance, and enhance patient satisfaction.

## Introduction

Facial abrasions are common injuries that can lead to the formation of scars, which not only affect the aesthetic appearance but may also cause psychological distress to patients [1,2]. Traditional wound-healing methods often result in suboptimal scar outcomes with issues such as hyperpigmentation, hypertrophy, and poor texture [3,4].

Drug-moist wound healing is based on the principle of maintaining a moist environment at the wound site. This approach can promote cell migration, proliferation, and angiogenesis, thereby facilitating wound repair and reducing scar formation. Photoelectric therapy, including laser and light-emitting diode (LED) therapies, has been increasingly used in scar treatment. Laser therapy can target specific chromophores in the skin, such as melanin and hemoglobin, to improve scar color and texture. LED therapy can stimulate cellular metabolism and collagen synthesis, promoting tissue repair [5,6].

The combination of drug-moist wound healing and photoelectric therapy may offer a synergistic effect in the treatment of facial abrasion scars [7]. However, there is limited research on the efficacy of this combined approach. Therefore, this study was conducted to investigate the therapeutic effects of drug-moist wound healing combined with photoelectric therapy in the healing of facial abrasion scars.

## Materials and methods

Materials and methods

Patients

Based on the results of the comprehensive pre-experiment, the experience of previous studies, and statistical analysis, it was ultimately determined that 20 patients per group would be the sample size for this study. A total of 40 patients with facial abrasion scars were recruited from the dermatology department of our hospital between January 2023 and December 2024. Patients were randomly assigned to one of two groups using a random number table: the saline group (control group) and the combined treatment group (observation group). The observation group included 20 patients (10 males and 10 females), aged five to 80 years, with a mean age of 24.45 ± 18.14 years. The control group also consisted of 20 patients (10 males and 10 females), aged five to 67 years, with a mean age of 24.90 ± 21.55 years. No significant differences were observed between the two groups in terms of age, sex, or other baseline characteristics (P > 0.05), indicating good comparability. The inclusion criteria were as follows: (1) patients aged between five and 80 years; (2) facial abrasions within three days of injury; (3) willingness to participate in the study and sign the informed consent form. The exclusion criteria were as follows: (1) patients with a history of allergic reactions to the drugs or photoelectric devices used in the study; (2) patients with severe underlying diseases such as diabetes, autoimmune diseases, or cancer; (3) patients who had received previous scar treatment within three months. This study was approved by the Ethics Committee of Huakang Dermatology Hospital (Approval No. 20230101), and all participants provided informed consent.

Treatment Methods

Saline and iodophor solution group (control group): The wounds were gently cleaned with normal saline to remove debris. A sterile cotton swab or saline-soaked gauze was used to carefully wipe the wound surface and remove any residual tissue debris or foreign particles.

Subsequently, the wound was disinfected with iodophor solution and covered with iodophor-soaked gauze. Dressing changes were performed every two to three days until complete wound healing was achieved.

During each dressing change, the wound was assessed for signs of infection, exudate volume, and healing progression. Patients were instructed to implement appropriate physical sun protection measures to prevent post-inflammatory hyperpigmentation.

Combined treatment group (observation group): The patients in this group received both drug-moist wound healing treatment and photoelectric therapy.

The wound surface was first cleaned with 0.9% sodium chloride solution (NS), followed by localized irradiation using LED red light at a wavelength of 630 nm (Manufacturer: Xuzhou Blue Electronic Technology Co., Ltd., Jiangsu, China). The output intensity was set at 80%-100% (not exceeding 80% for pediatric patients), with an irradiation duration of 15-20 minutes per session (8-10 minutes for children). The distance between the LED panel and the wound surface was maintained at 20-30 cm, and adjustments to distance and height were made as needed based on individual sensitivity to light, heat, and skin reactivity to prevent adverse effects. Protective eye masks were provided to all patients during irradiation to ensure ocular safety. Treatments were administered once daily, and the total treatment course lasted 10-15 days.

Immediately after each LED session, moist wound healing therapy was initiated using human epidermal growth factor gel (Trade name: Yifu; Manufacturer: Guilin Huanuo Gene Drug Co., Ltd., Guilin, China; Specification: 20 g/tube), applied in a layer approximately 1 mm thick, and continued until complete epithelialization and scab detachment.

For patients exhibiting persistent erythema following scab removal, adjunctive pulsed dye laser (PDL) therapy was administered at a wavelength of 595 nm (Manufacturer: Cynosure; Agent: Saironglong Medical Technology Co., Ltd., Beijing, China), with energy density set at 5-7 J/cm², pulse duration of 0.45-1.5 ms, and spot diameter of 5-7 mm. Treatments were repeated every 1-1.5 months as clinically indicated.

Assessment Indicators

Duration of wound healing: The period from the initiation of treatment to complete epithelialization of the wound was recorded for each patient.

Scar appearance: The scar appearance was evaluated using the Manchester Scar Scale (MSS) at three months and six months after the completion of treatment [8]. The MSS assesses three aspects of scar appearance: color, texture, and overall appearance, with a total score ranging from 0 to 15 (lower scores indicate better scar appearance). The use of the MSS scale had been approved by the author Beausang E, and was cited accordingly.

Patient-reported outcomes: A self-administered questionnaire was used to assess patient-reported outcomes, including pain during treatment, satisfaction with scar appearance, and overall quality of life related to the scar at six months after treatment. The questionnaire used a Likert scale from 1 (very dissatisfied) to 5 (very satisfied).

Statistical analysis

The data were analyzed using SPSS version 27.0 statistical software (IBM Corp., Armonk, NY). Measurement data are presented as mean ± standard deviation (mean ± SD), and intergroup comparisons were conducted using the independent samples t-test. A p-value less than 0.05 was considered statistically significant.

## Results

Duration of wound healing

The wound healing time in the combined treatment group was significantly shorter than that in the control group (t = 2.65, P < 0.05) (Table 1).

**Table 1:** Duration of wound healing between groups (mean ± SD, days). T-test.

	Control group (n = 20)	Observation group (n = 20)	t-values	P-value
Duration of wound healing	15.85 ± 3.13	13.45 ± 2.56	2.65	<0.001

Scar appearance

Manchester Scar Scale Scores at Three Months

At three months post treatment, the MSS scores in the combined treatment group were significantly lower than those in the control group (t = 4.59, P < 0.05), indicating improved scar appearance (Table 2).

**Table 2:** Manchester Scar Scale (MSS) scores for the assessment of scar appearance at three months post treatment (mean ± SD). T-test.

	Control group (n = 20)	Observation group (n = 20)	t-values	P-value
MSS scores	6.1 ± 1.21	3.85 ± 0.93	6.59	<0.001

Manchester Scar Scale Scores at Six Months

**Figure 1:**
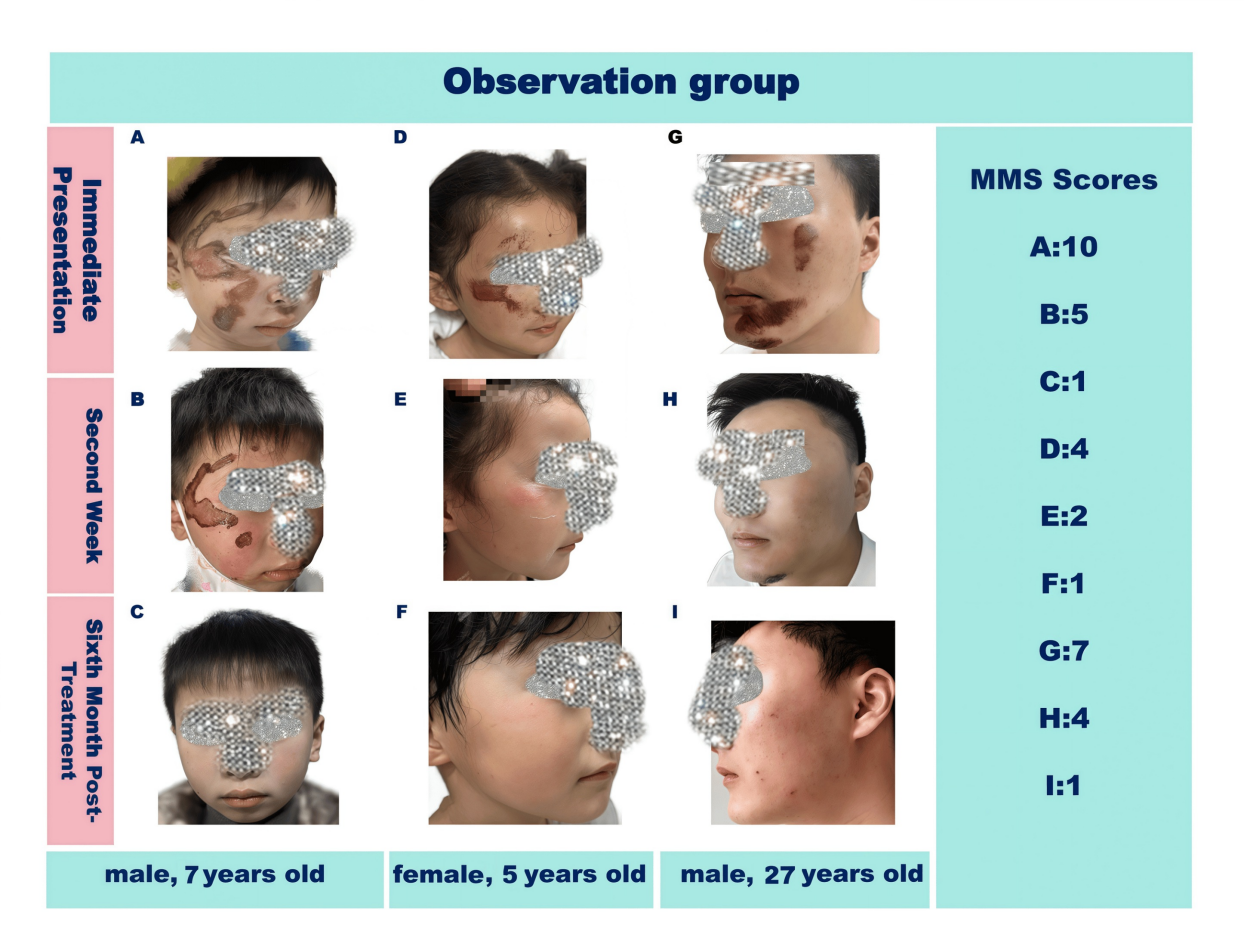
Facial abrasions in the combined therapy group.

The improvement in scar appearance was more evident at the six-month follow-up (Figure 1). The combined treatment group continued to exhibit the lower MSS scores. The differences between the groups were statistically significant (t = 7.48, P < 0.05) (Table 3).

**Table 3:** Manchester Scar Scale (MSS) scores for the assessment of scar appearance at six months post treatment (mean ± SD). T-test.

	Control group (n = 20)	Observation group (n = 20)	t-values	P-value
MSS scores	3.9 ± 0.91	1.75 ± 0.55	9.03	<0.001

Patient-Reported Outcomes

At six months after treatment, the patients in the combined treatment group reported significantly higher satisfaction with scar appearance and overall quality of life related to the scar compared to the control group (t = 6.87, 4.43, and 4.07, P < 0.05). They also reported less pain during treatment. The results are shown in Table 4.

**Table 4:** Questionnaire results using a Likert scale administered at six months post treatment (mean ± SD). T-test. The total score range of the Likert scale is from 1 to 5.

Groups	Control group (n = 20)	Observation group (n = 20)	t-values	P-value
Satisfaction with scar appearance	2.65 ± 0.99	4.50 ± 0.69	6.87	<0.001
Overall quality of life related to scar	3.25 ± 1.16	4.55 ± 0.61	4.43	<0.001
Pain during treatment observation	3.40 ± 0.68	4.25 ± 0.64	4.07	<0.001

## Discussion

The results of this study demonstrated that the combination of drug-moist wound healing and photoelectric therapy was significantly more effective than saline and iodophor solution blank therapy in accelerating the healing of facial abrasion scars and enhancing their aesthetic outcomes. Facial abrasions, often resulting from trauma or accidents, can lead to persistent scarring, pigmentation changes, and textural irregularities, which may have psychological and social implications for patients. Therefore, identifying an effective treatment regimen is crucial for optimal recovery [9-11].

Drug-moist wound healing is based on the principle that a moist environment promotes more rapid and efficient tissue regeneration compared to dry healing conditions. This approach establishes a favorable microenvironment conducive to cell proliferation, migration, and extracellular matrix deposition. In this study, human epidermal growth factor gel was incorporated into the drug-moist wound healing protocol, where it plays a critical role by enhancing wound repair through multiple biological mechanisms [12,13].

It promotes cell proliferation, stimulating keratinocytes, fibroblasts, and endothelial cells. Keratinocytes are key for re-epithelialization, and the gel signals them to divide, quickening new epithelial tissue formation. Fibroblast proliferation, enhanced by the gel, helps build a stable matrix with collagen and elastin. Endothelial cell growth is essential for wound angiogenesis.

The gel also facilitates cell migration. It creates a chemical gradient that attracts keratinocytes from the wound edges to the center, and aids fibroblasts in migrating to deposit the extracellular matrix, organizing healing tissue.

Moreover, it regulates extracellular matrix deposition. Fibroblasts, influenced by the gel's growth factors, produce proper amounts of matrix components, ensuring a healthy scar that can handle stress and looks normal [14-16].

Finally, it accelerates re-epithelialization by promoting keratinocyte growth and migration. This forms a protective barrier, preventing infection. Overall, the gel contributes to multiple wound repair aspects, leading to faster and more effective healing.

In parallel, LED therapy, particularly using red and near-infrared wavelengths, plays a complementary role by modulating cellular activity. LED photons penetrate the dermal layers and are absorbed by mitochondrial chromophores, such as cytochrome c oxidase, leading to enhanced adenosine triphosphate (ATP) production and reactive oxygen species signaling. This bioenergetic boost stimulates fibroblasts, increases collagen and elastin synthesis, and accelerates tissue remodeling. As a result, the scar becomes softer, flatter, and more closely resembles the surrounding healthy skin in both texture and appearance [17,18].

Additionally, photoelectric therapy contributed to improved scar outcomes through multiple biologically active mechanisms. The PDL, operating at a wavelength typically around 585-595 nm, selectively targets oxyhemoglobin within the microvasculature of hypertrophic or erythematous scar tissue. This selective photothermolysis leads to thermal damage of abnormal blood vessels, thereby reducing scar redness, vascularity, and inflammation, common features of immature scars. Over time, repeated treatments can result in a more even skin tone and less noticeable scarring [19,20].

The observed superiority of the combined therapy likely stems from a synergistic interaction between these two modalities. The hydrated wound bed established by drug-moist wound healing may improve the optical transmission of light energy during photoelectric therapy, allowing deeper and more uniform penetration of laser and LED light into the target tissues. Conversely, the anti-inflammatory and pro-regenerative effects of photoelectric therapy may enhance the quality of tissue formed under moist conditions, further supporting epithelial advancement and reducing the likelihood of adverse scarring.

Despite these promising findings, several limitations must be acknowledged. First, the sample size of the study was relatively small, which reduces statistical power and limits the ability to detect subtle differences between groups or apply the results broadly across diverse populations. Second, the follow-up period was limited, making it difficult to assess the long-term stability of scar improvement, including potential recurrence of redness or texture changes months after treatment cessation. Longer observational periods would provide valuable insights into the durability of the therapeutic effects.

In conclusion, the integration of drug-moist wound healing and photoelectric therapy represents a promising advancement in the management of facial abrasion scars. By leveraging the strengths of both biological and technological interventions, this combination addresses multiple aspects of the wound healing cascade, offering a comprehensive strategy for improved functional and cosmetic recovery.

## Conclusions

In conclusion, drug-moist wound healing combined with photoelectric therapy is an effective treatment approach for facial abrasion scars. It can significantly shorten the wound healing time, improve scar appearance, and enhance patient satisfaction. Future studies with larger sample sizes and longer follow-up periods are needed to confirm these findings and to further optimize the treatment protocol.

## Appendices

Manchester Scar Scale (MSS) scoring criteria

1. Pigmentation

0 points: The color is exactly the same as that of the surrounding normal skin.

1 point: Slight color change, slightly darker or lighter than the surrounding skin.

2 points: Moderate hyperpigmentation or hypopigmentation, with a noticeable difference from the surrounding skin.

3 points: Marked color change, significantly different from the surrounding skin.

4 points: Highly uneven color, with obvious areas of hyperpigmentation and hypopigmentation mixed.

5 points: Abnormal skin changes such as lichenification or verrucous hyperplasia occur.

2. Vascularity

0 points: No visible abnormal blood vessels.

1 point: Only a few dilated blood vessels are visible at the edge of the scar.

2 points: Blood vessels extend to the center of the scar, but the distribution is not extensive.

3 points: The blood vessels are relatively abundant and cover most of the scar surface.

4 points: Blood vessels are clustered in masses or nodules.

5 points: There are obvious vascular tumors.

3. Consistency

0 points: The scar is soft, with the same texture as the surrounding normal skin, and can be freely pinched.

1 point: Slightly harder than the surrounding skin, but can still be pinched relatively easily.

2 points: Significantly harder, and pinching is somewhat difficult.

3 points: The texture is hard and can hardly be pinched.

4 points: The scar shows contracture, restricting the normal movement of local skin or joints.

5 points: Severe contracture, resulting in obvious deformity or functional impairment.

Total score

The total score of the Manchester Scar Scale (MSS) is obtained by adding the scores from the above three aspects. The total score ranges from 0 to 15 points. The higher the score, the more severe the abnormality of the scar.

Self-administered questionnaire for patient-reported outcomes

Title: Patient-Reported Outcomes After Treatment

Instructions: This questionnaire is designed to gather your personal experiences and opinions regarding your treatment and its outcomes. Please answer each question as honestly as possible. All your responses will be kept strictly confidential.

We are interested in your experiences six months after the treatment. For each statement below, please choose a number from the Likert scale that best represents your level of agreement or your experience. The scale ranges from 1 (very dissatisfied) to 5 (very satisfied).

1. Pain during treatment

Please rate your level of pain during the treatment period.

01. Very dissatisfied (The pain was extremely severe and significantly affected my daily life)

02. Dissatisfied (The pain was quite bothersome and had a notable impact on my activities)

03. Neutral (The pain was tolerable and did not greatly disrupt my normal life)

04. Satisfied (The pain was mild and did not cause much interference)

05. Very satisfied (I experienced little to no pain during the treatment)

2. Satisfaction with scar appearance

Think about the appearance of your scar six months after the treatment. Rate your satisfaction with it.

01. Very dissatisfied (The scar is highly visible, disfiguring, and has a negative impact on my self-esteem)

02. Dissatisfied (The scar is noticeable, and I am not happy with how it looks)

03. Neutral (The scar is visible but does not cause me significant distress in terms of appearance)

04. Satisfied (The scar is relatively inconspicuous, and I am okay with its appearance)

05. Very satisfied (The scar is hardly visible, and I am very pleased with its appearance)

3. Overall quality of life related to the scar

Consider how the scar has influenced your overall quality of life in general, including your physical, emotional, and social aspects. Rate your overall feeling.

01. Very dissatisfied (The scar has severely restricted my physical activities, caused a lot of emotional distress, and affected my social life)

02. Dissatisfied (The scar has had a negative impact on my daily life, making me feel self-conscious and limiting some activities)

03. Neutral (The scar has a minor impact on my quality of life and does not significantly disrupt my normal routine)

04. Satisfied (The scar has little impact on my overall quality of life, and I can carry on with my daily activities as usual)

05. Very satisfied (The scar has no negative impact on my quality of life, and I feel completely normal in all aspects)

Thank you for taking the time to complete this questionnaire. Your responses will help us improve our treatment methods and patient care.
